# Iron acquisition strategies in pathogenic fungi

**DOI:** 10.1128/mbio.01211-25

**Published:** 2025-05-20

**Authors:** Kathryn Takemura, Vanessa Kolasinski, Matteo Del Poeta, Nathalia Fidelis Vieira de Sa, Ashna Garg, Iwao Ojima, Maurizio Del Poeta, Nivea Pereira de Sa

**Affiliations:** 1 Institute of Chemical Biology and Drug Discovery, Stony Brook, New York, USA; 2 Department of Microbiology and Immunology, Stony Brook University 273107, Stony Brook, New York, USA; 3 Division of Infectious Diseases, School of Medicine, Stony Brook University 12301, Stony Brook, New York, USA; 4 Veterans Affairs Medical Center, Northport, New York, USA; The Ohio State University, Columbus, Ohio, USA

**Keywords:** iron acquisition, siderophores, fungal infections, iron metabolism, iron-binding proteins, antifungal therapy

## Abstract

Iron plays a crucial role in various biological processes, including enzyme function, DNA replication, energy production, oxygen transport, lipid, and carbon metabolism. Although it is abundant in the Earth’s crust, its bioavailability is restricted by the insolubility of ferric iron (Fe³^+^) and the auto-oxidation of ferrous iron (Fe²^+^) in oxygen-rich environments. This limitation poses significant challenges for all organisms, including fungi, which have developed intricate mechanisms for iron acquisition and utilization. These mechanisms include reductive iron uptake, siderophore production/transport, and heme utilization. Fungi employ a variety of enzymes—such as ferric reductases, ferroxidases, permeases, and transporters—to regulate intracellular iron levels effectively. The challenge is heightened for pathogenic fungi during infection, as they must compete with the host’s iron-binding proteins like transferrin and lactoferrin, which sequester iron to restrict pathogen growth. This review delves into the iron acquisition strategies of medically important fungi, emphasizing the roles of reductive iron uptake and siderophore pathways. Understanding these mechanisms is vital for enhancing our knowledge of fungal pathogenesis and developing effective treatments. By targeting these iron acquisition processes, new antifungal therapies can be formulated more effectively to combat fungal infections.

## INTRODUCTION

Iron is essential for various biological functions, including metabolism, DNA replication, transcription, and energy production through respiration (1). However, while iron is vital, soluble ferrous iron (Fe²^+^) is also easily oxidized and potentially cytotoxic (2). At elevated levels, iron can be extremely detrimental to cellular growth and viability, causing nonspecific oxidation and damaging nucleic acids, lipids, and proteins (3, 4). This necessitates that organisms carefully balance iron acquisition with regulation to prevent toxic accumulation (3). To manage this, microorganisms, including fungi, developed iron uptake and storage strategies. These systems have been extensively characterized in the fungus *Saccharomyces cerevisiae* (5, 6). Broadly, the uptake of iron can be split into reductive and non-reductive pathways; the reductive uptake system relies on the extracellular reduction of ferric salts and chelates, which are then taken up by a high-affinity, ferrous-specific oxidase/permease complex, while the non-reductive system employs siderophores to scavenge iron from extracellular sources (7, 8). Additionally, several pathogenic fungi have developed methods to scavenge iron from heme, further facilitating iron uptake in the human host (9, 10). This review explores the iron acquisition strategies in medically significant fungi and how these strategies may be exploited in drug development.

## IRON ENVIRONMENT OF THE HOST

Iron is vital for both humans and microbial pathogens, yet its availability within the human body is tightly controlled to prevent infections (11). The majority of the iron in a healthy adult human circulates as hemoglobin in erythrocytes (~2,500 mg), some is stored as ferritin in hepatocytes and macrophages (~1,000 mg), a limited quantity of ferric iron is bound to transferrin (~3 mg) in the plasma, and the rest (~497 mg) is distributed among proteins such as ferroproteins and myoglobin among heart, muscles, and other organs (12–16). Heme, the iron-binding component of hemoglobin, acts not only as a cofactor but also as a signaling molecule for critical cellular processes, such as iron regulation, gas sensing, electron transfer, cell cycle progression, mitophagy, apoptosis, and managing oxidative stress (17–19).

Iron homeostasis is tightly regulated in the human body. When iron levels within the cells or extracellular iron stores are low due to physiological or pathological conditions such as inflammation, the hormone hepcidin, which is mainly produced by hepatocytes, is suppressed by FPN1 upregulation, enhancing iron absorption from the diet (20). In contrast, elevated iron levels stimulate hepcidin production by FPN1 downregulation, which reduces absorption by blocking the release of iron (both heme and non-heme) from enterocytes and macrophages (14, 21, 22). In the lungs, alveolar macrophages scavenge excess iron from inhaled particles or iron from plasma, limiting its availability (23–25).

The host employs intricate mechanisms to limit microbial access to iron, creating an environment that is less favorable for pathogen growth (26). This iron sequestration by the host is a crucial component of the host’s defense strategy as well as response to infection. Nutritional immunity—a process by which the availability of iron is highly restricted in the host—has been reported in vertebrates and invertebrates in response to infection, as discussed in detail by Hood and Skaar (26). Inflammatory signals, such as cytokines and chemokines, promote the synthesis of iron-binding proteins that will further sequester any extracellular free iron and encourage iron retention within host cells, effectively reducing the supply of this essential nutrient to invading microbes (1, 27–29).

**Fig 1:**
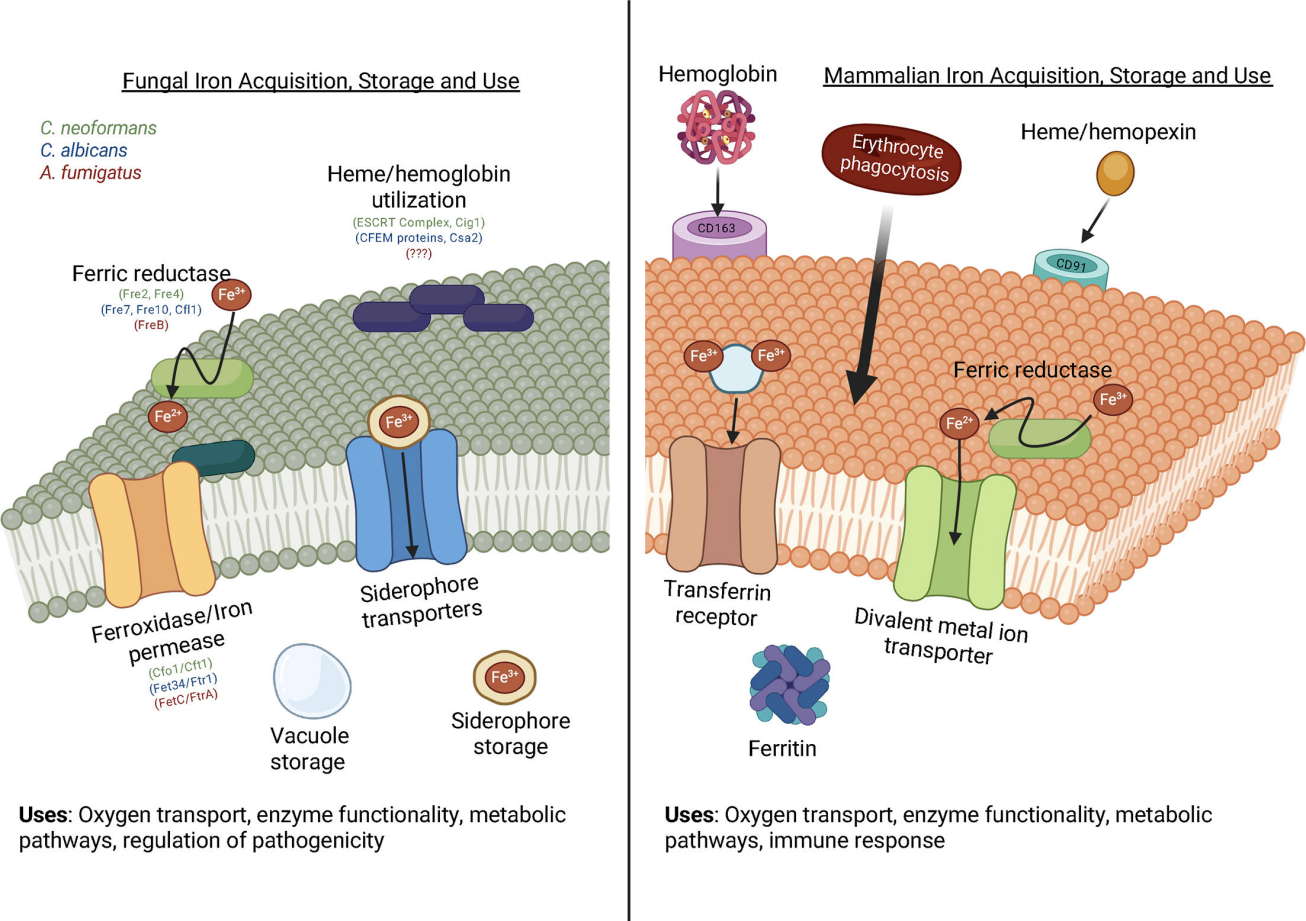
Comparison of iron acquisition, storage, and use by select pathogenic fungi and mammals. Pathogenic fungi have evolved methods of iron acquisition to facilitate growth in the host, including host-heme utilization, siderophore iron scavenging, and the reductive iron assimilation pathway, which includes ferric reductase, iron permease, and ferroxidase. Fungi store iron in vacuoles or specific storage siderophores. Mammals primarily obtain iron through dietary sources, but recycling strategies include senescent erythrocyte endocytosis and heme/hemoglobin recycling through CD91 and CD163, respectively. Mammals regulate iron through transferrin and ferritin, which can prevent iron from being used by microbes, although fungal siderophores have been shown to be able to scavenge iron from transferrin.

While iron sequestration can suppress pathogen proliferation, excessive iron accumulation in the intracellular compartment may disrupt the physiological balance, leading to potential tissue damage, thus releasing intracellular iron and fueling inflammatory conditions (30). To overcome the iron-limited conditions imposed by the host, pathogens have developed sophisticated mechanisms to secure iron, as detailed below. In Fig. 1, we summarize the iron uptake by fungal and host mammalian cells.

## BRIEF OVERVIEW OF FUNGAL IRON UPTAKE STRATEGIES

### Reductive iron uptake

Reductive iron assimilation is an essential mechanism employed by many organisms, including fungi, to extract iron from their environment, where it typically exists in the insoluble ferric (Fe³^+^) state (31, 32). Under aerobic conditions and neutral pH, ferric iron forms insoluble salts, posing a challenge for organisms (33). To address this issue, fungi use the reductive iron uptake pathway, which facilitates the reduction of ferric iron to its more physiologically available ferrous form (Fe^2+^) (31). This pathway predominantly relies on membrane-bound ferric reductases that catalyze the reduction of Fe³^+^ to Fe²^+^ at the cell surface (34).

In fungi, ferric reductases are an essential component of the reductive iron acquisition strategy. *S. cerevisiae* is a particularly well-studied case, with most focus being on Fre1p and Fre2p (Fig. 1) (35). Located in the plasma membrane, these flavocytochromes facilitate the electron transfer needed to reduce Fe³^+^ (7, 31), using NADPH or NADH as electron donors (36, 37). Both Fre1p and Fre2p are vital for iron reduction in conditions of iron scarcity, although Fre1p exhibits a broader substrate specificity that allows it to reduce not only Fe³^+^ but also other metals, such as copper (7, 38). Together, these reductases form the frontline of the reductive pathway, ensuring that iron is made available in the Fe²^+^ form for subsequent transport.

After ferric iron is reduced to the more soluble ferrous form, Fe²^+^ is transported across the plasma membrane. In many fungi, this transport is facilitated by high-affinity iron permeases working in conjunction with a multicopper ferroxidase (39), which re-oxidizes Fe²^+^ back to Fe³^+^ before it is transported intracellularly (40). This re-oxidation step is essential to prevent the buildup of free Fe²^+^ intracellularly, as excess Fe²^+^ can react with oxygen to generate reactive oxygen species via the Fenton reaction, potentially causing cellular damage (2). Ultimately, ferric ions are internalized through the high-affinity iron permease (4, 41). By effectively coordinating the reduction, transport, and reoxidation processes, fungi can mitigate the toxic effects of Fe²^+^ while securing the iron needed for their growth and metabolic functions (31). The reductive iron uptake pathway is tightly regulated based on the intracellular iron levels. While regulation and sensing are outside of the scope of this review, these processes are comprehensively covered by Gupta and Outten (42).

### Nonreductive iron uptake

**Fig 2:**
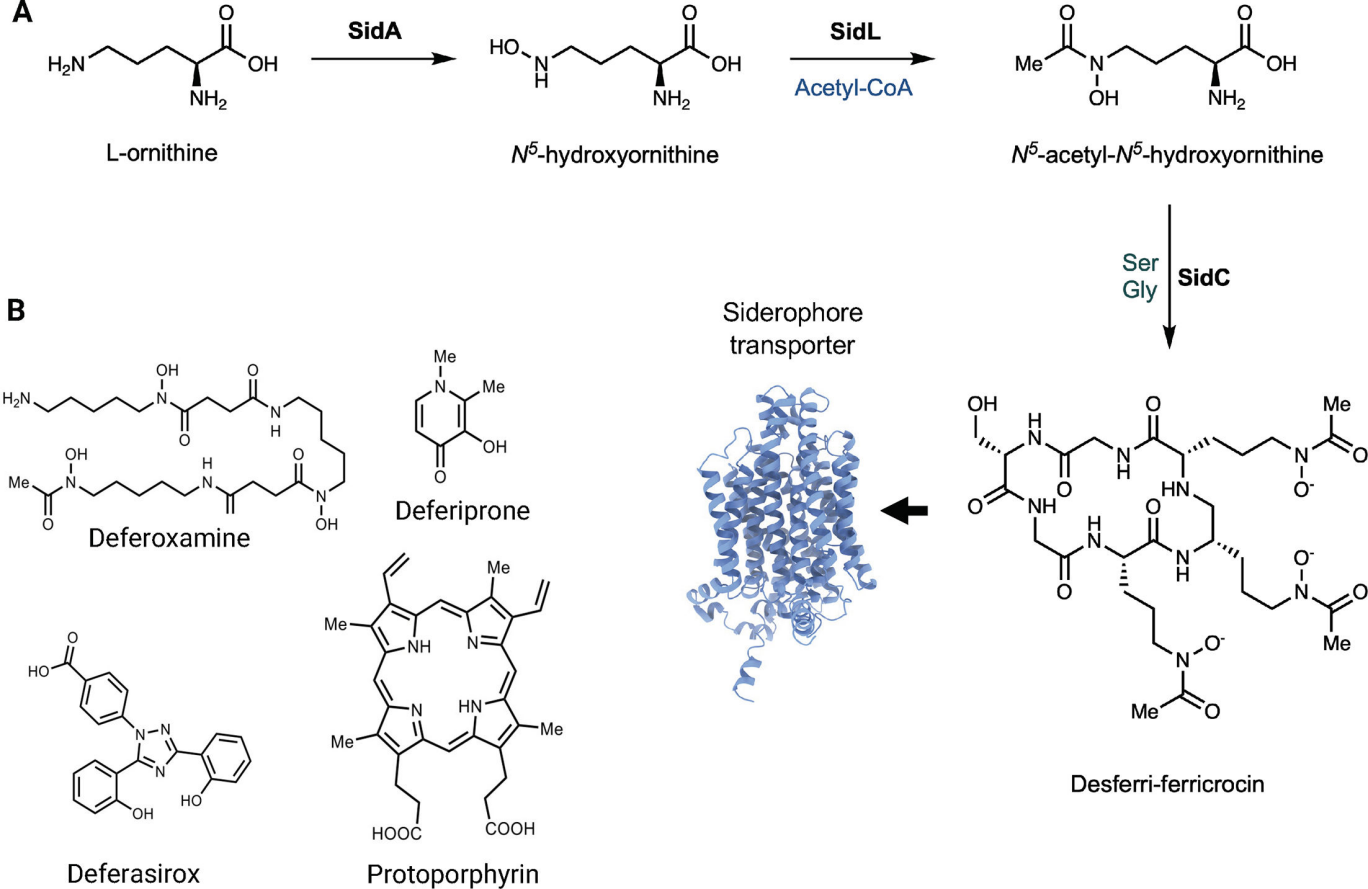
(**A**) General hydroxamate siderophore synthesis pathway. L-ornithine is hydroxylated by SidA, followed by acetylation by SidL. The peptide is synthesized by peptide synthetase SidC, yielding, in this example, desferri-ferricrocin. (**B**) Chemical structures of key iron-chelating agents and related compounds: deferoxamine (also called desferrioxamine B), a trihydroxamic acid siderophore; deferiprone, a bidentate hydroxypyridinone chelator; deferasirox, a tridentate chelator; and protoporphyrin, a precursor in heme biosynthesis.

Most fungi biosynthesize siderophores, which are small organic molecules ranging from 100 to 500 Da that exhibit a remarkable affinity and specificity for ferric iron. These fungal siderophores have dissociation constants ~10^−29^ M, indicating an affinity that far exceeds that of any other biologically relevant chelator (32). Almost all fungi possess a non-reductive uptake system specifically designed for siderophore-iron chelates (43). Iron bound to siderophores is transported inside the fungal cell by siderophore transporters. Interestingly, although some fungi do not produce siderophores, nearly all fungi express siderophore transporters, which can also recognize siderophores produced by other fungal species or even bacteria. Approximately 500 distinct compounds have been identified as siderophores, categorized into three main types: catecholates, hydroxamates, and carboxylates, with hydroxamates being the most common in fungi (44, 45). The majority of fungal siderophores fall within the hydroxamate category (Fig. 2A), derived from the amino acid L-ornithine (46). Additionally, fungal siderophores can be classified into four structural families: rhodotorulic acid, fusarinine, coprogen, and ferrichrome (24).

The uptake of iron using the non-reductive pathway is crucial for fungi, particularly pathogenic species that must navigate host-imposed iron restrictions detailed above. Siderophores enable iron uptake where it is typically limited due to iron sequestration by the host through transferrin and/or lactoferrin, which are present in the human respiratory tract fluids (47–50). In addition, siderophores are pivotal for virulence during several fungal infections, as inhibition of their synthesis or transport significantly reduced disease in murine models (51).

The biosynthesis of hydroxamate siderophores in fungi begins with the *N*5-hydroxylation of the amino acid L-ornithine (Fig. 2A). This initial step is performed by ornithine *N*5-oxygenase, which was first identified as the product of the Sid1 gene in *Ustilago maydis* (52). This enzyme has also been recognized as SidA in *Aspergillus nidulans* and *Aspergillus fumigatus* and as dffA in *Aspergillus oryzae* (53, 54). In *A. fumigatus*, SidA catalyzes the conversion of L-ornithine into *N*5-hydroxyornithine. This intermediate is then acetylated by *N*5-hydroxyornithine acetyltransferase (SidL), followed by processing by non-ribosomal peptide synthetases (NRPSs) (55). Among these, SidC plays a vital role in *A. fumigatus* by linking the hydroxamate groups and facilitating the assembly and cyclization of the siderophore backbone (Fig. 2A) (45). These NRPS enzymes are conserved across various pathogenic fungi, suggesting a common mechanism for siderophore production among different fungal species (56, 57).

After synthesis, siderophores are secreted into the extracellular space to scavenge ferric iron. Specific transporters mediate this secretion. In *A. fumigatus*, an ABC transporter known as SidF is responsible for the secretion of triacetylfusarinine C (TAFC), enabling the fungus to scavenge iron from its environment, where iron is predominantly found in the insoluble ferric form. *Neurospora crassa* and *Fusarium graminearum* employ similar mechanisms for siderophore secretion, underscoring the conserved nature of this pathway among filamentous fungi (45).

Siderophores bind to ferric iron, forming Fe^3+^-siderophore complexes that are recognized by siderophore transporter proteins on the surface of fungal cells (Fig. 1). Siderophore transporters belong to the ARN/SIT subfamily of the major facilitator superfamily (MFS). This superfamily is characterized by its multiple transmembrane domains, which enable the transport of substrates across the plasma membrane (58). These transporters typically operate through a proton-coupled mechanism, involving the recognition and binding of Fe^3+^-siderophore complexes followed by conformational changes that facilitate the transport of iron into the cell (59).

Early studies of *S. cerevisiae* established a model for the uptake mechanisms of xenosiderophore-iron chelates, particularly through the Arn/Sit transporters (6, 60). These transporters—Arn1, Arn2/Taf1, Arn3/Sit1, and Arn4/Enb1—exhibit distinct specificities for various bacterial and fungal xenosiderophores, including enterobactin, ferrirubin, ferrirhodin, ferrichrome, ferrichrome A, triacetylfusarinine C, and ferrioxamine B. The uptake process has been demonstrated through the trafficking of ferrioxamine B Arn3/Sit1 and ferrichrome-bound Arn1 transporters (61).

Each transporter shows specificity for different groups of fungal and/or bacterial siderophores, although variations in the specificity of individual transporters have been observed among different strains of *S. cerevisiae*. For example, Arn1p is responsible for transporting ferrichrome and related hydroxamate siderophores, such as ferrichrome A, ferricrocin, ferrirhodin, ferrichrysin, and ferrirubin. Additionally, Arn1p recognizes coprogen and displays a slight activity toward triacetylfusarinine C (62–64).

For Arn1, two conformational changes occur with ligand binding, the first after one molecule of extracellular ferrichrome is endocytosed and bound to Arn1 (which has two binding sites) at the endosomal compartment, causing re-localization of the protein to the plasma membrane. The second conformational change is initiated by the binding of a second molecule of ferrichrome, leading to endocytosis and internalization of the ferrichrome-bound Arn1 to the cytosol. Finally, ferrichrome is dissociated from Arn1, degraded, and iron is released, while Arn1 is recycled (51). The release of iron from the siderophore can occur through either reduction or hydrolysis. In the case of reduction, the ferric iron is reduced to ferrous iron, for which the siderophore has a significantly decreased affinity, and iron release allows for the recycling of the siderophore. In the case of hydrolysis, esterase enzymes (such as EstB for TAFC and SidJ for FsC in *A. fumigatus*) hydrolyze the Fe^3+^-siderophore complex to liberate Fe^3+^ for cellular utilization (65, 66).

Similarly, Arn3/Sit1 is also displayed to the plasma membrane in the presence of its substrate ferrioxamine B, whereas it is sorted to the vacuolar pathway in its absence for degradation, facilitating the release of Fe^3+^ iron (59, 67). The movement of Sit1 into the lumen of the multivesicular body relies on Rsp5-mediated ubiquitylation and the clathrin adaptor protein Gga2 (68). The abundance of plasma membrane transporters, including metal transporters, is controlled according to physiological needs, including substrate concentration. A common feature of regulating the transporters on the plasma membrane is to trigger their internalization and sorting to the endosome/lysosome degradation pathway. In response to specific signals, such as a change in nutrient availability, transporters stored in intracellular compartments can be sorted to the plasma membrane (69). Other examples of fungal iron transporters that belong to the MFS include Fet3, a multicopper oxidase that collaborates with the iron transporter Ftr1 to facilitate the import of Fe^3+^-siderophore (31). In *S. cerevisiae*, a family of such transporters is encoded by the *SMF1-3* genes (70).

The regulation of the Fe^3+^-siderophore acquisition pathway is closely tied to the iron concentration within the cell. When iron levels are low, transcriptional mechanisms activate the biosynthesis, secretion, and uptake of siderophores to satisfy the iron needs of the fungal cell. In *A. fumigatus*, the GATA-type transcription factor SreA (Table 1) acts to repress siderophore production when iron is plentiful, thereby preventing unnecessary synthesis of siderophores (71, 72). In contrast, under conditions of iron deficiency, HapX enhances siderophore production while simultaneously suppressing processes that consume iron, ensuring that all available iron is utilized effectively. This regulatory mechanism allows fungi to maintain a balance between iron acquisition and storage, which is particularly crucial in environments such as the human body, particularly in the alveolar spaces, where iron availability is very limited due to host defense strategies such as intracellular iron sequestration by macrophages (23, 71).

**TABLE 1:** Enzymes associated with iron homeostasis in the mitochondria of human fungal pathogens

Enzyme	Fungal pathogen	Function	Role of iron	References
Cytochrome c oxidase (Complex IV)	*Candida albicans*, *A. fumigatus*, and *Cryptococcus neoformans*	Catalyzes electron transfer from cytochrome c to oxygen in the electron transport chain, driving ATP synthesis.	Iron is part of heme groups essential for electron transfer.	(73)
Catalase	*C. albicans*, *A. fumigatus*, and *C. neoformans*	Breaks down hydrogen peroxide into water and oxygen to protect against oxidative stress.	Iron is part of the active site, catalyzing the reaction.	(74)
Superoxide dismutase	*C. albicans*, *A. fumigatus*, and *C. neoformans*	Converts superoxide radicals into oxygen and hydrogen peroxide to reduce oxidative damage	Iron is an essential cofactor for Fe-SOD.	(75)
Ribonucleotide reductase	*C. albicans*, *A. fumigatus*, and *C. neoformans*	Converts ribonucleotides into deoxyribonucleotides, a key step in DNA synthesis.	Iron forms a stable tyrosyl radical at the enzyme’s active site.	(15, 76)
Iron-sulfur cluster proteins, examples: NADH dehydrogenase (Complex I), aconitase	*C. albicans*, *A. fumigatus*, and *C. neoformans*	Facilitate electron transfer and metabolic reactions.	Iron-sulfur clusters are integral cofactors for these proteins.	(77)
Nitric oxide reductase	*C. albicans*, *A. fumigatus*, and *C. neoformans*	Detoxifies nitric oxide, aiding in immune evasion during infection.	Uses a heme-iron center to catalyze the reduction of nitric oxide.	(78)
Heme oxygenase	*C. albicans*, *A. fumigatus*, and *C. neoformans*	Breaks down heme into biliverdin, free iron, and carbon monoxide, aiding in iron recycling.	Requires heme iron for catalytic activity.	(15)
Ferroxidases	*C. albicans*, *A. fumigatus*, and *C. neoformans*	Oxidizes Fe(II) to Fe(III) to facilitate iron uptake.	Essential for iron acquisition and homeostasis.	(32, 79, 80)
SreA/Sre1 (iron-responsive transcription factor)	*A. fumigatus* and *C. neoformans*	Regulates iron uptake and storage, controlling iron homeostasis under low iron conditions.	Responds to iron availability and impacts fungal virulence.	(7, 81)
Succinate dehydrogenase (Complex II)	*C. albicans*, *A. fumigatus*, and *C. neoformans*	Functions in the TCA cycle and electron transport chain.	Contains iron-sulfur clusters for electron transfer.	(82)
Laccases (multicopper oxidases)	*A. fumigatus* and *C. neoformans*	Plays a role in pigmentation, oxidative stress resistance, and virulence.	Indirectly influenced by iron availability, crucial for activity	(83)

### Heme utilization

Due to its availability in the host, heme has driven fungi to develop specialized mechanisms for acquiring it, especially in environments that are otherwise iron limited. For example, the iron content of the blood aside from heme is quite low, but pathogens can tap into the plentiful heme reserves to acquire iron.

In contrast to other acquisition strategies, the mechanisms of iron acquisition from heme in human fungal pathogens are less understood. However, iron acquisition from hemoglobin was studied in *Candida albicans*, which has specific receptors, such as Rbt5 and Rbt51, for the uptake of hemoglobin across the plasma membrane (84, 85). Hemoglobin binds to Rbt5 and is internalized through an endocytic process (79). Once inside the vacuole, hemoglobin is likely broken down or hydrolyzed to release heme, with the heme oxygenase (Table 1) Hmx1 playing a key role in degrading heme to liberate iron for cellular use (86). Overexpressing *C. albicans* Rbt51 in *S. cerevisiae* enables the latter to grow in the presence of hemoglobin as a sole iron source, possibly through the vacuolar ATPase and the endosomal sorting complex required for transport (ESCRT) machinery, which is responsible for directing monoubiquitinated membrane proteins to the vacuole (87, 88). In fact, *C. albicans* mutants lacking components of the ESCRT complex showed impaired iron acquisition from hemoglobin, though their ability to utilize iron from the siderophore ferrichrome remained unaffected (87). Other receptors for hemoglobin uptake have been described, including Wap1/Csa1, Csa2, and Pga7, which possess a characteristic extracellular membrane protein domain that is shared among fungi but not mammalian cells (89, 90).

In *Cryptococcus neoformans*, studies have shown that the ESCRT-I component Vps23 contributes to the regulation of iron acquisition, capsule, and melanin production, both of which are crucial for this pathogen’s virulence (87). In addition, Vps22 (ESCRT-II) and Snf7/Vps20 (ESCRT-III) are related to the use of heme (91). The extracellular mannoprotein Cig1 and the transcription factor Rim101 are required for iron acquisition from heme at physiological pH (Fig. 1). The loss of Cig1 also resulted in attenuated virulence in a mouse model of cryptococcosis (92).

Heme-binding activity in *Histoplasma capsulatum* has been detected on the cell surface, allowing the pathogen to acquire iron from heme, siderophores, and iron-binding proteins like transferrin (93). This process involves the secreted enzyme gamma-glutamyltransferase (Ggt1) and a glutathione-dependent ferric reductase (GSH-FeR) (94). Ggt1 plays a key role in virulence, alongside specific siderophores (73).

## IRON UPTAKE AND STORAGE OF PATHOGENIC FUNGI

### Cryptococcus neoformans

*Cryptococcus neoformans*, a pathogenic fungus responsible for cryptococcosis, presents a considerable risk to individuals with weakened immune systems, as it can lead to severe meningoencephalitis (74). *C. neoformans* employ a range of mechanisms to acquire iron, as iron regulates the synthesis of crucial virulence factors, including melanin and the polysaccharide capsule (75). Iron is acquired using both high- and low-affinity iron permeases, as well as reductive strategies involving melanin and laccase (Table 1) (76). Additionally, the fungus utilizes iron reductases and secretes 3-hydroxyanthranilic acid as a reductant. This versatile pathogen can internalize xenosiderophores and heme iron. The complex formed by the Cft1 permease and Cfo1 ferroxidase (Fig. 1) is vital for reductive iron uptake and for seizing iron from transferrin, significantly enhancing virulence in mouse models of cryptococcosis (75).

The reductive iron uptake pathway is the most well-studied mechanism for iron acquisition in *C. neoformans*, specifically targeting the host’s iron storage proteins like transferrin. The genome of *C. neoformans* contains multiple putative ferric reductases. For example, Fre4 is essential for proper melanin production, and its absence results in heightened susceptibility to antifungal treatments. Meanwhile, Fre2 is critical for virulence in mouse models of infection. The ferroxidase Cfo1 requires localization to the plasma membrane for its optimal activity, a process that is supported by cAMP signaling (77). In terms of iron transport, two high-affinity transporters, Cft1 and Cft2 (Fig. 1), play essential roles in virulence. Although Cft2 is not required for growth in low-iron environments, it may serve as a vacuolar iron transporter, facilitating the mobilization of stored iron reserves to the cytoplasm. Cft1, on the other hand, is crucial for virulent growth within the brain of infected mice, indicating that transferrin may be a particularly significant source of iron in this tissue. The expressions of both *CFT1* and *CFT2* are regulated by a cAMP-dependent protein kinase, with the transcription factor Cir1 exerting opposing regulatory effects on their expression (77).

Multiple transcription factors manage iron homeostasis in response to the host environment. The principal regulator is Cir1, which orchestrates the transcriptional response to iron scarcity by exerting both positive and negative influences on various virulence-related pathways, including those involving cAMP/PKA, calmodulin, and MAP kinase signaling. Cir1 is essential for capsule formation, growth at 37°C, and overall virulence, while also affecting iron-dependent processes such as ergosterol biosynthesis and glycolysis. Notably, Cir1 inhibits the reductive iron uptake pathway (78).

A secondary regulatory layer involves the transcription factors HapX and Rim101. HapX enhances the expression of siderophore transporters and represses electron transport under low-iron conditions while also inducing Cir1 and Rim101 expression. The absence of HapX leads to a slight but significant decrease in virulence. The nuclear localization of Rim101 is regulated by the Rim/Pal signaling pathway in response to changes in pH, contributing to the activation of genes involved in various iron uptake pathways and capsule formation (80).

The non-reductive iron uptake pathway in *C. neoformans* consists of the siderophore-mediated iron acquisition. *C. neoformans* is unable to produce siderophores but uses xenosiderophores synthesized by other microorganisms as an iron source. In *C. neoformans*, six siderophore transporters were identified (Sit1-6) (51). However, only Sit1 has been characterized, and the functions and roles in the virulence of the remaining paralogs Sit2-Sit6 have not yet been demonstrated (51, 81). In addition, Sit1 was not required for virulence in a murine model of cryptococcosis but was found to influence melanin formation, cell wall integrity, and growth at 37°C during iron limitation (81). Under low iron conditions, the transcription factor Cir1 binds to the promoter of *SIT1* and *SIT2*, whereas HapX binds to SIT3 to positively regulate their expression. In contrast, under high iron levels, the protein binds to SIT4 and SIT6 to repress gene expression (82). However, how the siderophore gene expression translates into iron acquisition is poorly understood in this organism, which cannot rely on its own siderophores to transport iron through its transporters.

### Aspergillus fumigatus

*Aspergillus fumigatus* is a widely distributed saprophytic fungus and stands out as the most prevalent airborne fungal pathogen affecting humans. The clinical manifestations caused by *A. fumigatus* vary, ranging from allergic reactions to severe invasive diseases collectively known as aspergillosis, particularly impacting immunocompromised individuals (83).

This fungus secretes fusarinine C (FsC) and triacetylfusarinine C to capture Fe³^+^ iron, which it subsequently absorbs via siderophore iron transporters. *A. fumigatus* harbors five putative siderophore iron transporters (MirB, MirC, MirD, Sit1, and Sit2) that are transcriptionally inhibited via SreA in the presence of high iron availability in the extracellular environment (95–98). In addition to external siderophores, *A. fumigatus* synthesizes two intracellular ferrichrome-type siderophores, ferricrocin and hydroxyferricrocin, which are essential for Fe^3+^ iron storage, distribution, and management within the cell. These siderophores aid in the handling of iron within the hyphae and conidia. Iron detoxification and storage occur in the vacuole via the CccA vacuolar transporter, although it remains uncertain if this stored iron can be recycled. Moreover, iron is also imported into mitochondria for utilization in biosynthetic pathways through the importer MrsA. In this organelle, iron is essential for the function of many enzymes in fungal pathogens (Table 1).

In the face of iron scarcity, *A. fumigatus* experiences significant transcriptional changes driven by two critical transcription factors operating within a negative feedback loop: the GATA-factor SreA and the bZip-factor HapX (96). When iron is abundant in the extracellular environment, SreA suppresses iron uptake mechanisms (Table 1), including reductive Fe^2+^ iron assimilation and siderophore-mediated uptake of Fe³^+^, to mitigate potential toxicity. Conversely, under conditions of iron deprivation extracellularly, HapX inhibits iron-consuming pathways such as heme biosynthesis and respiration to conserve this vital nutrient (63, 96). Additionally, HapX stimulates the production of the ribotoxin AspF1 and siderophores by ensuring an adequate supply of the precursor ornithine (99).

The effects of disrupting SreA and HapX are context dependent: SreA deficiency primarily impacts growth in iron-sufficient environments, while HapX deficiency hinders growth during iron starvation (71). Importantly, the absence of HapX, rather than SreA, diminishes the virulence of *A. fumigatus* in murine models, highlighting the significance of iron adaptation for pathogenicity. Both extracellular and intracellular siderophore production are consistently essential for the virulence of this fungus. Recent research has also illuminated the role of the sterol regulatory element-binding protein SrbA, which is vital for adapting to iron starvation (24). This links the regulation of iron metabolism to ergosterol biosynthesis, azole drug resistance, and responses to hypoxic conditions.

In a murine model of aspergillosis, MirB is essential for virulence, making it a promising target for antifungal therapies since this transporter is absent in mammalian hosts (100). The siderophore system is also critical for the intracellular survival of *A. fumigatus* following phagocytosis by alveolar macrophages. Deficiencies in siderophore biosynthesis can significantly influence the host’s immune response, highlighting the importance of this mechanism in the pathogen’s ability to evade immune defenses. MirB transporter uptake Fe³^+^-TAFC complex. Structural studies of MirB have demonstrated its localization in vesicles, cycling between the cytoplasm and the plasma membrane, where it is concentrated at the hyphal tips. MirB can also bind to ferricrocin and coprogen as well as TAFC but not ferrichrysin (101). When Fe³^+^-TAFC binds to MirB, it initiates a series of conformational changes in the transporter protein that are essential for the transport process. Initially, MirB adopts a specific conformation that allows it to recognize and bind the Fe³^+^-TAFC complex at the extracellular side of the membrane (101). Upon binding, MirB closes off the extracellular side while opening the intracellular side. This transition enables the release of Fe³^+^ into the cytoplasm while simultaneously preventing the backflow of iron or the ferrichrome complex (24). This mechanism exemplifies the “alternating access” transport process, where the transporter alternates between different conformations, exposing binding sites alternately to the outside and inside of the cell.

Following the uptake of TAFC and ferrichrome-iron complexes through siderophore iron transporters, hydrolysis occurs within the cytosol, and two esterases, EstB and SidJ, have been identified as key players in this process (4, 102, 103). Specifically, EstB acts on TAFC, while SidJ is tailored for ferricrocin. However, they both seem to facilitate the release of iron, although they are not essential for the hydrolysis of siderophores (102, 103).

In a recent study by Michels et al. (104), researchers explored the ability of *A. fumigatus* to utilize heme as an iron source during infection. Their investigation centered on lung hemorrhage, a characteristic feature of invasive aspergillosis, revealing a correlation between increased lung hemoglobin levels and elevated iron linked to heme within the first 3 days of infection. Significantly, the introduction of heme during infection exacerbated both the severity of the disease and the fungal burden in the lungs. Pre-culturing germinated conidia with heme resulted in greater lung damage and increased fungal loads upon infection. These findings collectively suggest that *A. fumigatu*s can indeed utilize heme during infection. However, questions remain regarding the mechanisms involved in heme acquisition, particularly its relationship to iron uptake through siderophore production (104).

### Candida albicans

Candidiasis, caused by the dimorphic fungus *Candida albicans*, poses a significant threat as an opportunistic infection in humans. This organism is part of the normal human gut microbiota but can transition from a harmless commensal to a pathogenic form under specific conditions. *C. albicans* has evolved mechanisms to thrive in environments with fluctuating iron availability, such as the iron-poor bloodstream and the iron-rich gastrointestinal tract. To effectively manage iron limitations within the human host, *C. albicans* has established a sophisticated regulatory system for iron acquisition and storage (105).

Although *C. albicans* does not synthesize its siderophores, it can scavenge iron that is bound to xenosiderophores. Mutation studies in the only identified siderophore-iron transporter, *sit1*Δ/Δ, do not significantly impact virulence in murine models of systemic candidiasis; however, these mutations hinder the fungus’s ability to inflict damage on *ex vivo* human keratinocyte tissue (59). The Sit1/Arn1 transporter allows *C. albicans* to utilize xenosiderophores such as ferricrocin, ferrichrysin, ferrirubin, coprogen, and triacetylfusarinine C. Given its role as a human commensal, *C. albicans* likely encounters these xenosiderophores from bacteria inhabiting mucosal or gastrointestinal niches. The Sit1/Arn1 transporter is essential for effective invasion of reconstituted epithelial tissues, which serve as a model for human oral mucosa, although it does not appear to influence virulence in systemic candidiasis models, indicating that siderophore-mediated iron uptake may not be crucial during bloodstream infections (59).

*C. albicans* utilizes the reductive iron uptake pathway to extract iron from the host’s ferritin and transferrin stores, while also acquiring free iron (106, 107). The fungus binds to host ferritin through the surface adhesion protein Als3, particularly in its hyphal form (107). Besides its role in iron acquisition, Als3 is important for biofilm formation and adherence to host cells. Deletion of the *ALS3* gene reduces virulence in surface infection models, though its impact is less significant in the context of systemic infection (107, 108).

Once ferrous iron (Fe^2+^) is solubilized, it is re-oxidized to the ferric form by the multicopper oxidase CaFet3, which depends on copper provided by the intracellular copper transporter Ccc2 (105, 109). A *fet3*Δ/Δ mutant shows impaired growth in iron-limited conditions, although its virulence is only slightly reduced compared to the wild-type strain (110). Ferroxidase iron is subsequently internalized through the high-affinity iron permease CaFtr1, which is crucial for survival in iron-limited media and plays a significant role in virulence. While *C. albicans* has another putative high-affinity iron permease, CaFtr2, it is not essential for survival or pathogenicity, and its transcriptional regulation differs from that of CaFtr1 (111).

The heme-iron uptake system in *C. albicans* is regulated by the transcription factor Hap1 and relies on the expression of the Common in Fungal Extracellular Membrane (CFEM) hemophore system (Fig. 1), which includes proteins such as *RBT5*, *RBT51*, *CSA1*, *CSA2*, and *PGA7* (112). These hemophores are vital for internalizing and transporting heme bound to proteins such as hemoglobin and human serum albumin from the external environment to the plasma membrane through the CFEM hemophore cascade (113). Although the transport systems for heme have not been fully characterized in fungi, *C. albicans* can take up heme as an intact molecule, which is then degraded by the intracellular heme oxygenase CaHmx1p. This degradation is essential for utilizing heme iron for growth (89).

The heme uptake system in *C. albicans* operates independently of the reductive iron uptake system, as evidenced by mutant strains lacking *CaCCC2* or *CaFTR1* still being able to utilize heme as an iron source (113). Moreover, the heme uptake process may share characteristics with the uptake of small, hydrophobic molecules such as oxysterols. For instance, *S. cerevisiae* absorbs sterols under low oxygen and heme-deficient conditions, relying on the cell wall mannoprotein Dan1p and ABC transporters Aus1p or Pdr11p. In *C. albicans*, the GPI-anchored cell surface mannoprotein CaRbt5p is implicated in heme uptake, while a related protein, CaRbt51p, enhances heme uptake when expressed in *S. cerevisiae*.

The heme acquisition system is dependent on a cascade of extracellular CFEM hemophores, both soluble and anchored to the cell wall or membrane, as well as the endosomal sorting complex required for transport pathway and membrane ferric reductase-like proteins (111). The CFEM gene *RBT5* is one of the most strongly induced genes in experimental animal models of infection, and Rbt5-specific antibodies are prevalent in the serum of patients recovering from candidemia, underscoring the significance of this heme-acquisition pathway during infection (112). The three secreted CFEM proteins involved in hemoglobin-iron acquisition—Csa2, Rbt5, and Pga7—are capable of extracting heme from hemoglobin and transferring it among themselves, indicating a heme transfer cascade across the cell envelope followed by internalization via endocytosis (113). The development of sophisticated means for heme acquisition may be due to the fact that this fungus is more hematogenous compared to other fungi, such as *C. neoformans* and *A. fumigatus*, which are less commonly found in the bloodstream compared to *C. albicans*.

## IRON ACQUISITION AS A TARGET FOR DRUG DEVELOPMENT

Current strategies for targeting pathogen iron acquisition encompass a variety of approaches, including heme analogs, iron chelation therapy, siderophore-drug conjugates, inhibitors of uptake systems and biosynthetic enzymes, vaccines against surface uptake components, and immunological methods aimed at influencing iron availability for pathogens (105, 114–116). Notably, focusing on biosynthetic enzymes for inhibitor development shows considerable promise, particularly for pathogens that utilize siderophores during host colonization.

Iron is crucial for the survival and growth of fungi, as it supports metabolic processes essential for their proliferation. Disrupting these iron acquisition mechanisms with chelators, which bind to iron and render it unavailable, can severely compromise fungal viability, leading to stunted growth or even cell death (116–118). In contrast, mammalian cells have evolved a more robust regulatory system for managing iron depletion than fungi, such as primarily stocking iron associated with ferritin, a protein that sequesters and releases iron in response to its fluctuating levels (14).

The potential of chelators in treating fungal infections is gaining traction, with recent studies highlighting the development of novel chelators and their combination with antifungal drugs. For instance, Helsel et al. (118) screened a range of metal chelating agents for their ability to inhibit the growth of *C. neoformans*. The compounds tested included general chelators, iron-specific chelators such as desferrioxamine B (Fig. 2B), and molecules that bind copper. Interestingly, while those targeting iron or copper did not inhibit fungal growth under the tested conditions, compounds that increased intracellular copper levels were found to be inhibitory. These active agents, identified as ionophores, exhibited fungicidal properties against *C. neoformans* (118). Even more recently, Liang et al. (119) reported an iron-chelating compound with picomolar activity against *C. neoformans*, further showcasing that targeting iron metabolism is a promising avenue in antifungal drug development.

Targeting iron acquisition through siderophores has also been reported in the literature. Siderophores, which are readily taken up by fungi of nearly all species, are an attractive target, but due to the incredibly high affinity of siderophores for Fe^3+^, designing a better small molecule chelator is not a tenable strategy. However, polymeric compounds have been used to exceed the affinity of fungal siderophores for iron, effectively inhibiting iron siderophore-based iron acquisition (120). Finally, by conjugating antifungal compounds to siderophores, compounds can be selectively delivered to fungal cells and then released upon siderophore hydrolysis. This “Trojan horse” strategy has been reported in fungi, bacteria, and some parasites (121–123).

While exploiting iron uptake mechanisms in pathogens holds significant therapeutic promise, caution is warranted since fungi can still obtain iron from siderophores used as chelators, such as deferoxamine. For example, patients with diabetic ketoacidosis or those undergoing dialysis are at increased risk for mucormycosis when treated with the bacterial siderophore deferoxamine (124, 125). However, chelators like deferiprone and deferasirox (Fig. 2B), which are not utilized by the fungus, have shown protective effects in mice with diabetic ketoacidosis and have been employed in clinical trials (126–128).

Additionally, toxic analogs of heme, such as non-iron metalloprotoporphyrins, utilize heme uptake systems for their inhibitory effects against bacterial pathogens and show potential efficacy against *C. neoformans* (Fig. 2B) (87). This approach may also prove useful for other fungal pathogens that rely on siderophores, such as *A. fumigatus* and *Fusarium oxysporum* (116, 117, 129, 130).

Finally, the potential for targeting iron acquisition functions in vaccine development has been demonstrated in various pathogens, including bacteria, *C. albicans*, and *Rhizopus oryzae*, which causes mucormycosis in patients with diabetic ketoacidosis (131–134). Notably, a monoclonal antibody against the ferritin-binding protein Als3 has been shown to interfere with iron acquisition in *C. albicans*, exhibiting fungicidal properties (132).

## CONCLUSIONS AND PERSPECTIVES

Understanding the differences and similarities between the host and fungal pathogens regarding iron homeostasis and uptake strategies is crucial for identifying novel therapeutic targets against pathogenic fungi. Fungi have developed highly specialized iron acquisition strategies that are essential for their survival and pathogenicity in both environmental and host settings. The secretion of siderophores, the exploitation of host iron-binding proteins, and the utilization of iron reduction mechanisms enable fungi to thrive even in iron-limited conditions. These processes not only support fungal growth but also enhance virulence by aiding fungi in evading the host’s immune defenses. Gaining insights into these intricate mechanisms provides valuable knowledge for the development of antifungal therapies.

Targeting fungal iron acquisition pathways presents significant therapeutic potential. By disrupting key components such as siderophore production, iron transporters, or iron reduction systems, novel antifungal drugs can effectively inhibit fungal growth and reduce infection severity. This approach is particularly timely given the rising threat of drug-resistant fungal strains. Future research should focus on refining these strategies to enhance specificity, minimize off-target effects, and improve overall treatment efficacy. As these strategies evolve, they hold promise for developing more effective antifungal therapies and addressing the urgent need to combat challenging fungal infections.
